# Global prevalence and burden of multidrug-resistant tuberculosis from 1990 to 2019

**DOI:** 10.1186/s12879-024-09079-5

**Published:** 2024-02-22

**Authors:** Hengliang Lv, Xin Zhang, Xueli Zhang, Junzhu Bai, Shumeng You, Xuan Li, Shenlong Li, Yong Wang, Wenyi Zhang, Yuanyong Xu

**Affiliations:** 1 Department of Epidemiology, School of Public Health, China Medical University, Shenyang, China; 2 Chinese PLA Center for Disease Control and Prevention, Beijing, China; 3 Changchun University of Chinese Medicine, Changchun, China; 4 Department of Epidemiology and Biostatistics, School of Public Health, Anhui Medical University, Hefei, China

**Keywords:** Global, Multidrug resistant tuberculosis, Prevalence, Disability-adjusted life years, Joinpoint regression

## Abstract

**Background:**

Tuberculosis(TB) remains a pressing public health challenge, with multidrug-resistant tuberculosis (MDR-TB) emerging as a major threat. And healthcare authorities require reliable epidemiological evidence as a crucial reference to address this issue effectively. The aim was to offer a comprehensive epidemiological assessment of the global prevalence and burden of MDR-TB from 1990 to 2019.

**Methods:**

Estimates and 95% uncertainty intervals (UIs) for the age-standardized prevalence rate (ASPR), age-standardized incidence rate (ASIR), age-standardized disability-adjusted life years rate (ASR of DALYs), and age-standardized death rate (ASDR) of MDR-TB were obtained from the Global Burden of Disease (GBD) 2019 database. The prevalence and burden of MDR-TB in 2019 were illustrated in the population and regional distribution. Temporal trends were analyzed by using Joinpoint regression analysis to calculate the annual percentage change (APC), average annual percentage change (AAPC) and its 95% confidence interval(*CI*).

**Results:**

The estimates of the number of cases were 687,839(95% UIs: 365,512 to 1223,262), the ASPR were 8.26 per 100,000 (95%UIs: 4.61 to 15.20), the ASR of DALYs were 52.38 per 100,000 (95%UIs: 22.64 to 97.60) and the ASDR were 1.36 per 100,000 (95%UIs: 0.54 to 2.59) of MDR-TB at global in 2019. Substantial burden was observed in Africa and Southeast Asia. Males exhibited higher ASPR, ASR of DALYs, and ASDR than females across most age groups, with the burden of MDR-TB increasing with age. Additionally, significant increases were observed globally in the ASIR (AAPC = 5.8; 95%*CI*: 5.4 to 6.1; *P* < 0.001), ASPR (AAPC = 5.9; 95%*CI*: 5.4 to 6.4; *P* < 0.001), ASR of DALYs (AAPC = 4.6; 95%*CI*: 4.2 to 5.0; *P* < 0.001) and ASDR (AAPC = 4.4; 95%*CI*: 4.0 to 4.8; *P* < 0.001) of MDR-TB from 1990 to 2019.

**Conclusions:**

This study underscored the persistent threat of drug-resistant tuberculosis to public health. It is imperative that countries and organizations worldwide take immediate and concerted action to implement measures aimed at significantly reducing the burden of TB.

**Supplementary Information:**

The online version contains supplementary material available at 10.1186/s12879-024-09079-5.

## Introduction

Tuberculosis (TB) is a highly contagious disease caused by the *Mycobacterium tuberculosis*(*Mtb*), primarily transmitted through the air [1]. It is characterized by a long incubation period, lack of early symptoms, and ease of transmission [2]. TB continued to be a significant global health concern, ranking as the second leading cause of infectious disease mortality, resulting in 1.4 million deaths in 2021 [3]. The expenses of TB diagnostic, treatment and prevention services in low and middle-income countries were estimated at US$ 5.4 billion in 2021 [1], which has brought heavy economic and social burden to the countries around the world. Nevertheless, estimated in 27 countries from the country-specific models, which suggested that there could be further increases in the incidence and deaths of TB [1].

Multidrug-resistant TB (MDR-TB), defined as TB caused by *Mtb* bacilli resistant to rifampicin and isoniazid, represents a major threat to global TB control [4]. In 2021, there were an estimated 450,000 cases of MDR-TB, representing a 3.1% increase from the previous year [1]. About 464,000 global cases of rifampicin-resistant TB, 78% of which were MDR-TB was noticed at global in 2019 [5], and approximately 25% of deaths related to TB can be attributed to antimicrobial drug resistance [6]. Relevant studies have shown that MDR-TB is a debilitating disease that can give rise to severe and secular physical [7], mental [8], and financial sequelae [9]. Although countries have been expanding diagnostic capacity, detecting more patients with MDR-TB over recent years, a large number of cases has still been reported in some countries of Central Asia and Eastern Europe, such as China, India, and Russia [1, 10, 11]. In addition, MDR-TB imposes a significant burden on healthcare systems, with treatment costs 20 times higher than those of drug-susceptible TB [12]. Given its impact, MDR-TB deserves increased attention and prioritization globally.

The continuous dissemination of MDR-TB poses one of the most challenging and urgent obstacles to global TB control efforts [13]. Enhanced surveillance and data collection are crucial for assessing the risk of MDR-TB transmission. Understanding the global prevalence of MDR-TB will be helpful in the optimal allocation of limited resources to control its spread. The aim was to provide epidemiological evidence to the department of health by describing the prevalence of MDR-TB globally, utilizing data from the Global Burden of Disease (GBD) study.

## Methods

### Data source

For the present study, we acquired the MDR-TB data from the GBD 2019 database, available at http://ghdx.healthdata.org/gbd-results-tool. The GBD 2019 database is widely recognized for its valuable, systematic, and comprehensive approach in collecting and analyzing epidemiological data, which provides standardized measures of incidence, prevalence, mortality, DALYs (disability-adjusted life years), and other indicators for 369 injuries and diseases across 204 countries and territories, spanning the years from 1990 to 2019. Statistical code used for GBD estimation is publicly available online from http://ghdx.healthdata.org/gbd-2019/code. Supplement 1 [14] provides some details on the methods used to model MDR-TB. The methodology, data inputs and processing used in GBD 2019 have been extensively described in other high-quality studies [14–16], related information of TB has been utilized to described previously as well [17, 18].

We concentrated on the differences of MDR-TB between sexes, ages, and regions. And the estimates and 95% uncertainty intervals (UIs) for the number of cases, age-standardized prevalence rate (ASPR), age-standardized incidence rate (ASIR), age-standardized disability-adjusted life years (ASR of DALYs), and age-standardized death rate (ASDR) of MDR-TB were extracted from GBD 2019.

### Disease defination and description

The section on drug-resistant TB in the GBD database primarily focuses on the following four aspects: (i) human immunodeficiency virus(HIV)/acquired immune deficiency syndrome(AIDS)-MDR TB without extensive drug resistance(abbreviated to MDR-TB throughout the other part of this study), (ii) HIV/AIDS-Extensive drug-resistant TB, (iii) MDR-TB, and (iv) Extensive drug-resistant TB. This study specifically focuses on the research of MDR-TB. Multidrug-resistant TB (MDR-TB), defined as TB caused by *Mtb* bacilli resistant to rifampicin and isoniazid [4]. The study provided a comprehensive description of MDR-TB by examining multiple disease indicators, encompassing prevalence, DALYs rate, and death rate in different population subgroups, including gender and age, globally in 2019, additionally, regarding regional distribution of MDR-TB, the number of cases, ASPR, ASR of DALYs, and ASDR were adopted to compare the differences in the burden of disease across countries and territories in 2019.

### Temporal trend analysis

Joinpoint regression analysis [19] was employed to analyze the temporal trends of ASIR, ASPR, ASR of DALYs, and ASDR from 1990 to 2019 at the global/region level. This method allows the identification of significant joinpoints, which are points indicating substantial changes in the trend. The analysis divides the trend into multiple subsegments, and the annual percentage change (APC) with a 95% confidence interval (*CI*) is calculated for each subsegment. Additionally, the average annual percentage change (AAPC) is used to summarize the overall change trends from 1990 to 2019. A positive APC/AAPC estimation along with the lower boundary of its 95%*CI* greater than zero indicates an upward trend during a specific period. Conversely, if the APC/AAPC estimation and the upper boundary of its 95%*CI* are both below zero, a declining trend is observed. If neither of these conditions is met, the trend is considered stable [20, 21]. 

### Statistical analysis

We utilized bar graphs to visually represent the disparities of MDR-TB among different sexes and age groups. World maps were employed to demonstrate the geographic variances in the age-standardized rate in 2019. Furthermore, to calculate the APC and AAPC, we employed Joinpoint regression software and line charts were employed to depict the disease trends from 1990 to 2019. All statistical analyses and visualizations were executed using R software (version 4.2.1) and Joinpoint regression software (version 4.9.1.0). We considered a *P* < 0.05 as significant.

## Results

### ASPR of MDR-TB

**Fig. 1:**
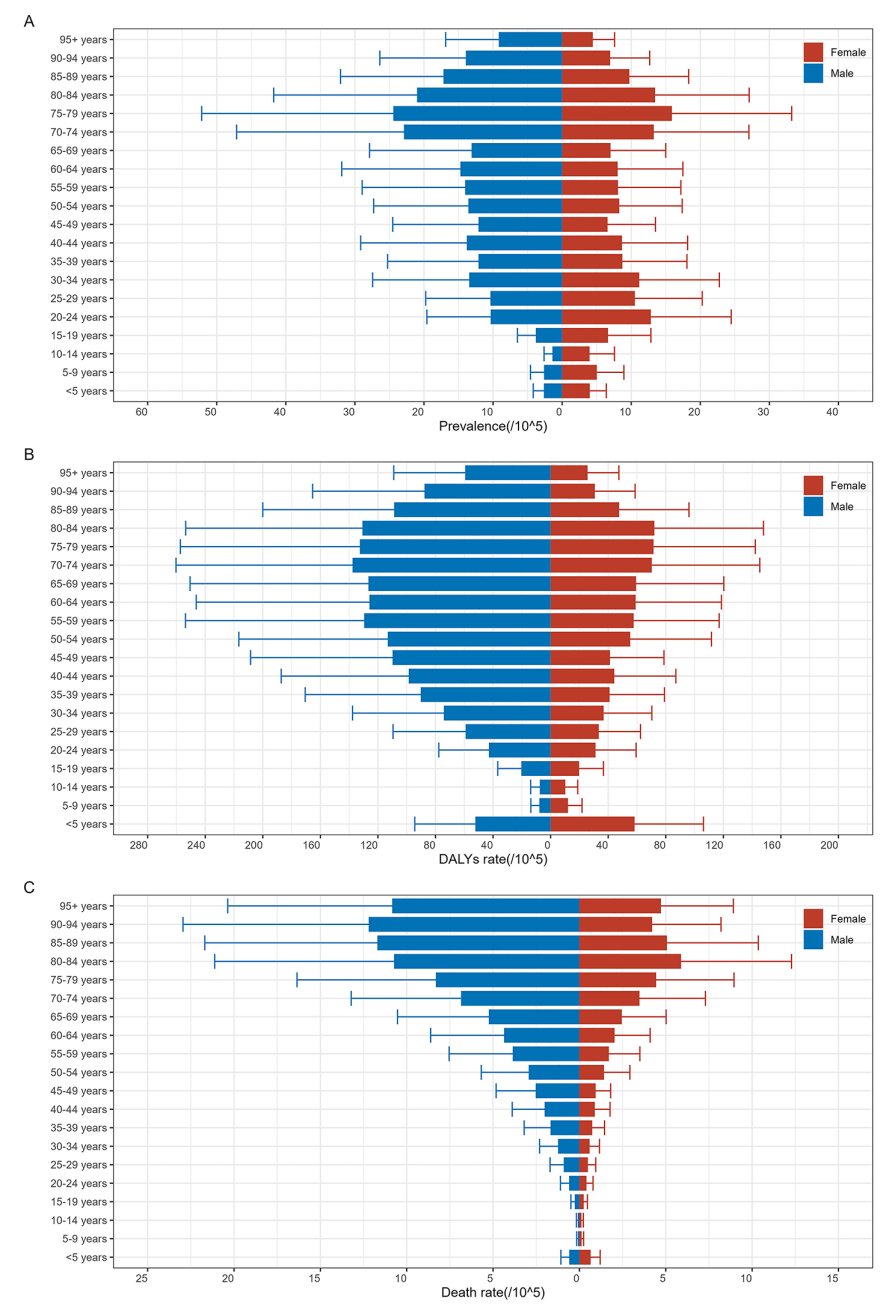
Age and sex distribution of ASPR **(a)**, ASR of DALYs **(b)** and ASDR of MDR-TB **(c)** of MDR-TB at global in 2019. ASPR:age-standardized prevalence rate; ASR of DALYs: age-standardized of Disability-adjusted life years rate; ASDR:age-standardised death rate

**Fig. 2:**
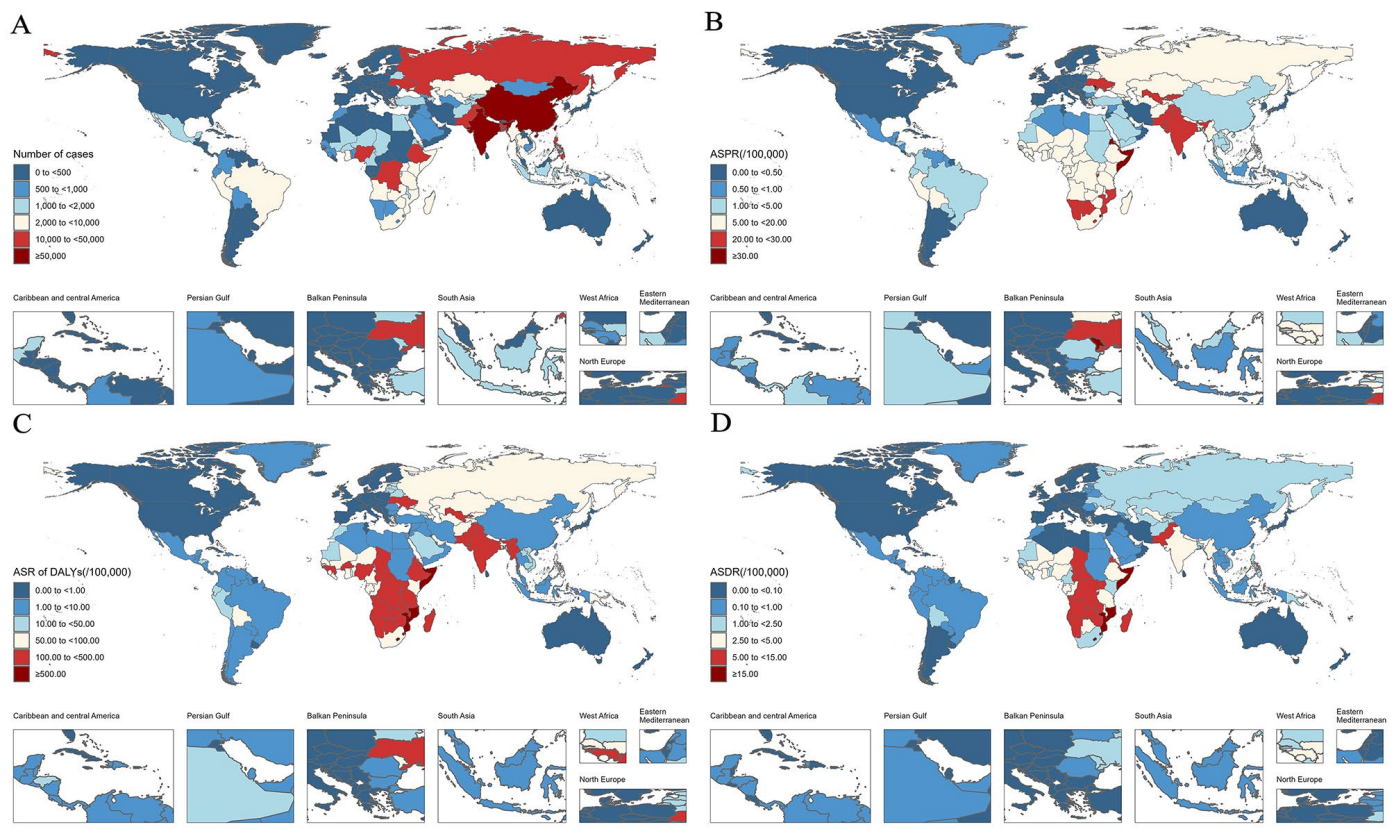
Number of case **(a)**, ASPR **(b)**, ASR of DALYs **(c)** and ASDR **(d)** of MDR-TB of MDR-TB by country in 2019. ASPR:age-standardized prevalence rate; ASR of DALYs: age-standardized of Disability-adjusted life years rate; ASDR:age-standardised death rate

**Fig. 3:**
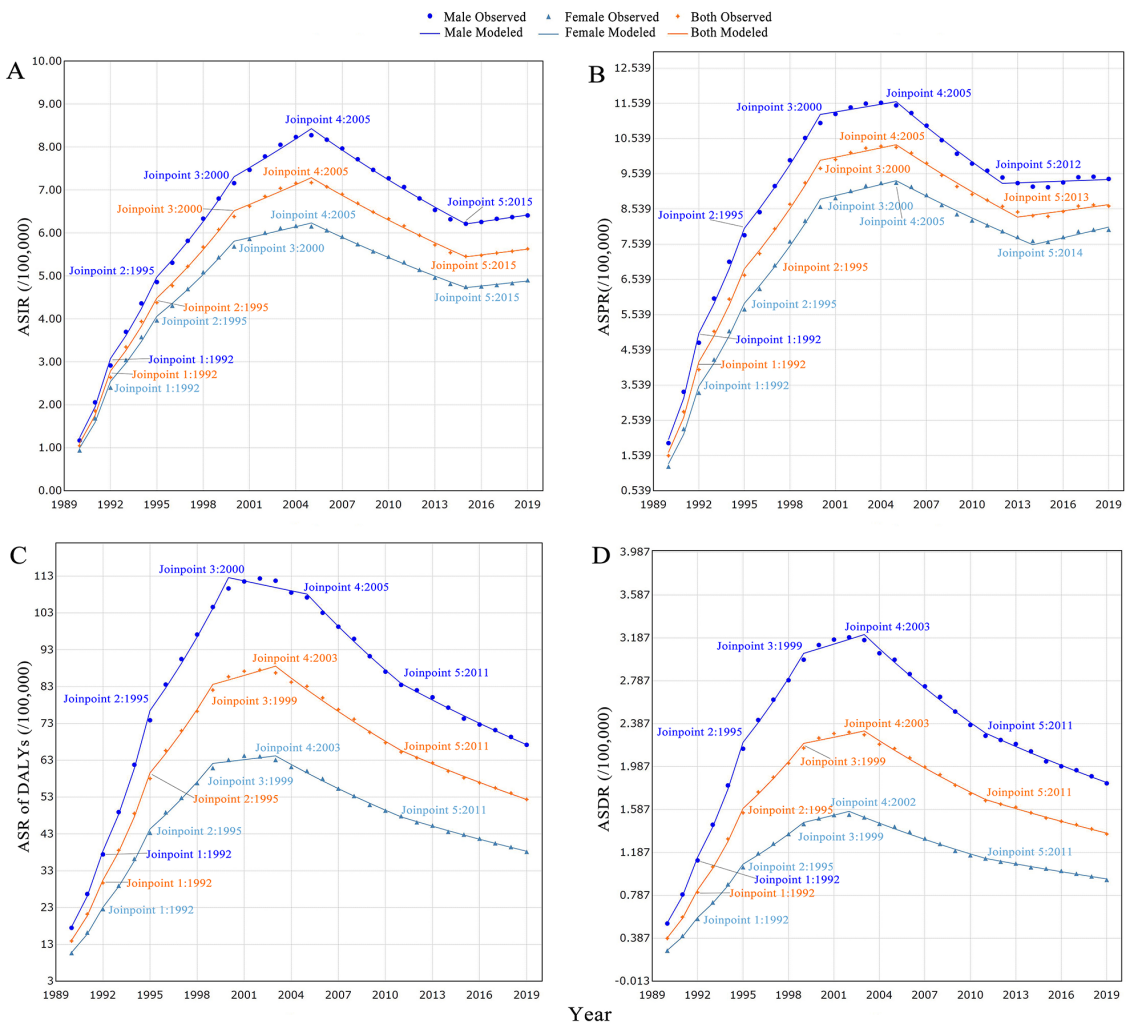
Joinpoint regression analysis of ASIR **(a)**, ASPR **(b)**, ASR of DALYs **(c)** and ASDR **(d)** of MDR-TB at the global from 1990 to 2019. ASIR:age-standardized incidence rate; ASPR:age-standardized prevalence rate; ASR of DALYs: age-standardized of Disability-adjusted life years rate; ASDR:age-standardised death rate

Based on the data extracted from the GBD 2019 database, some meaningful findings are listed below. The prevalence of MDR-TB was higher in men (9.39/100,000; 95% UIs: 4.99 to 16.79) compared to women (7.95/100,000; 95% UIs: 4.29 to 13.69). The highest prevalence was noted in the 75–79 years age group (19.74/100,000; 95% UIs: 8.20 to 39.86) (Fig. 1A), and males had a higher prevalence rate than females across most age groups (Fig. 1A). The number of MDR-TB was 687,839(95% UIs: 365,512 to 1223,262) at global in 2019, and Fig. 2A showed the top three cases were in India, China and Pakistan. The ASPR of MDR-TB was 8.26 per 100,000 population (95% UIs: 4.61 to 15.20) at global, and the Sub-Saharan Africa showed a higher burden than other continents, the country with the highest ASPR was Somalia (48.86/100,000; 95% UIs: 13.62 to 140.00), while Slovenia had the lowest ASPR (0.01/100,000; 95% UIs: 0.00 to 0.03) (Fig. 2B) across 204 countries in 2019. The global ASPR of MDR-TB showed a significant increasing trend from 1990 to 2019 (AAPC = 5.9, 95%*CI*: 5.4 to 6.4; *P* < 0.001) (Fig. 3B; Table 1), with the most enormous changes occurring during 1990–1992 (APC = 60.3; 95%*CI*: 52.3 to 68.7; *P* < 0.001). However, there was a decline in the trend between 2005 and 2013 (APC = -2.7; 95%*CI*: -3.0 to -2.5; *P* < 0.001), followed by a subsequent rise (Table 2).

**Table 1:** Global AAPCs in ASIR, ASPR, ASR of DALYs and ASDR of MDR-TB from 1990 to 2019

Measure	Sex		AAPC		95%CI			t		*P*
					Lower	Upper				
ASIR										
	Both		5.8		5.4	6.1		32.3		< 0.001
	Male		5.8		5.4	6.2		29.7		< 0.001
	Female		5.6		5.3	6.0		35.6		< 0.001
ASPR										
	Both		5.9		5.4	6.4		24.2		< 0.001
	Male		5.5		5.0	5.9		23.7		< 0.001
	Female		6.5		6.0	6.9		27.6		< 0.001
ASR of DALYs										
	Both		4.6		4.2	5.0		22.2		< 0.001
	Male		4.7		4.3	5.1		23.8		< 0.001
	Female		4.5		4.1	4.8		23.6		< 0.001
ASDR										
	Both		4.4		4.0	4.8		21.6		< 0.001
	Male		4.4		3.9	4.8		19.5		< 0.001
	Female		4.3		3.8	4.8		17.5		< 0.001

ASIR:age-standardized incidence rate; ASPR:age-standardized prevalence rate; ASR of DALYs: age-standardized of Disability-adjusted life years rate; ASDR:age-standardised death rate; AAPC:average annual percentage change; *CI*:confidence interval.

**Table 2:** Global APCs in ASIR, ASPR, ASR of DALYs and ASDR of MDR-TB from 1990 to 2019

Measure		APC	95% CI		t	*P*
			Lower	Upper		
ASIR						
	1990–1992	58.6	52.8	64.7	26.6	< 0.001
	1992–1995	17.2	14.9	19.5	17.6	< 0.001
	1995–2000	7.8	7.1	8.4	27.8	< 0.001
	2000–2005	2.3	1.8	2.7	10.7	< 0.001
	2005–2015	-2.9	-3.0	-2.7	-43.4	< 0.001
	2015–2019	0.8	-0.1	1.7	2.0	0.067
ASPR						
	1990–1992	60.3	52.3	68.7	19.9	< 0.001
	1992–1995	17.7	14.3	21.1	12.2	< 0.001
	1995–2000	7.7	6.8	8.6	19.5	< 0.001
	2000–2005	0.9	0.3	1.5	3.2	0.008
	2005–2013	-2.7	-3.0	-2.5	-24.6	< 0.001
	2013–2019	0.7	0.1	1.3	2.6	0.024
ASR of DALYs						
	1990–1992	46.9	42.1	51.9	24.7	< 0.001
	1992–1995	25.0	21.9	28.3	18.8	< 0.001
	1995–1999	8.8	7.4	10.3	13.9	< 0.001
	1999–2003	1.5	0.3	2.6	2.8	0.016
	2003–2011	-3.7	-3.9	-3.4	-28.3	< 0.001
	2011–2019	-2.8	-3.1	-2.5	-22.5	< 0.001
ASDR						
	1990–1992	46.3	41.6	51.1	25.2	< 0.001
	1992–1995	24.0	20.9	27.2	18.4	< 0.001
	1995–1999	8.4	7.0	9.8	13.5	< 0.001
	1999–2003	1.3	0.2	2.4	2.5	0.026
	2003–2011	-3.9	-4.2	-3.7	-30.7	< 0.001
	2011–2019	-2.5	-2.8	-2.3	-20.3	< 0.001

ASIR:age-standardized incidence rate; ASPR:age-standardized prevalence rate; ASR of DALYs: age-standardized of Disability-adjusted life years rate; ASDR:age-standardised death rate; AAPC:average annual percentage change; *CI*:confidence interval.

### ASIR of MDR-TB from 1990 to 2019

Joinpoint regression analysis was also conducted to analyze the annual age-standardized incidence rates (ASIR) of MDR-TB from 1990 to 2019. The main findings revealed a substantial upward trend in the ASIR of MDR-TB (AAPC = 5.8; 95%*CI*: 5.4 to 6.1; *P* < 0.001), which was consistent for both males (AAPC = 5.8; 95%*CI*: 5.4 to 6.2; *P* < 0.001) (Fig. 3A; Table 1) and females (AAPC = 5.6; 95%*CI*: 5.3 to 6.0; *P* < 0.001) (Fig. 3A; Table 1). The increasing trend was interrupted around 2005, followed by a downward trend from 2005 to 2015 (APC = -2.9; 95%*CI*: -3.0 to -2.7; *P* < 0.001) (Table 2).

### ASR of DALYs of MDR-TB

The age-standardized rate (ASR) of disability-adjusted life years (DALYs) for MDR-TB was 52.38 per 100,000 population (95%UIs: 97.60 to 22.64) in 2019. Males generally exhibited higher DALYs than females across most age groups. The most significant DALYs occurred in the 70–74 years age group for men, while for women, it was highest in the 80–84 years age group (Fig. 1B). Similar to the ASPR, the Sub-Saharan Africa exhibited a high burden of ASR of DALYs, Somalia had the highest ASR of DALYs (1010.92/100,000; 95%UIs: 230.54 to 2778.92), while Slovenia had the lowest ASR of DALYs (0.02/100,000; 95%UIs: 0.00 to 0.07) (Fig. 2C). From 1990 to 2019, the ASR of DALYs for both sexes demonstrated a year-by-year upward trend (AAPC = 4.6, 95%*CI*: 4.2 to 5.0; *P* < 0.001) (Fig. 3C; Table 1. The increasing trend was interrupted around 2003, followed by a downward trend from 2003 to 2011 (APC = -3.7, 95%*CI*: -3.9 to -3.4; *P* < 0.001)(Table 2), subsequently, the ASR of DALYs continued to decline until 2019.

### ASDR of MDR-TB

Across 204 countries and regions in 2019, the death rate of MDR-TB was higher in men (1.83/100,000; 95%UIs: 0.75 to 3.51) compared to women (0.93/100,000; 95% UIs: 0.37 to 1.81) (Fig. 1C). The most significant death rate occurred in the 90–94 years age group for men, while for women, it was highest in the 80–84 years age group (Fig. 1C). The age-standardized death rate (ASDR) ascribable to MDR-TB was 1.36 per 100,000 population (95% UIs: 0.54 to 2.59). The Sub-Saharan Africa showed a high burden of ASDR as well, and Somalia had the highest ASDR (31.90/100,000; 95% UIs: 7.12 to 88.59), while countries like Slovenia, Bermuda, and Andorra had the lowest ASDR (0.00/100,000; 95% UIs: 0.00 to 0.00) (Fig. 2D). Joinpoint regression analysis indicated a considerable rising trend in the global ASDR of MDR-TB from 1990 to 2019 (AAPC = 4.4, 95%*CI*: 4.0 to 4.8; *P* < 0.001) (Fig. 3D; Table 1). The increasing trend was interrupted around 2003, followed by a downward trend from 2003 to 2011 (APC = -3.9, 95%*CI*: -4.2 to -3.7; *P* < 0.001). Subsequently, the rate of increase slowed down from 2011 to 2019 (APC = -2.5, 95%*CI*: -2.8 to -2.3; *P* < 0.001) (Table 2).

## Discussion

In this study, we analyzed the data from GBD 2019 to describe the population and regional distribution of the burden of MDR-TB at the national and global levels in 2019. We also examined the temporal trends from 1990 to 2019. The main strengths of this study contain a large data base covering populations worldwide, a wide time span, and comprehensive geographic coverage.

We observed that males had higher prevalence, DALYs, and death rate than females in most age groups. This finding was consistent with the Global TB reports provided by the WHO [1, 4, 5], which reported a higher number of TB cases among men. Men tend to be more closely associated with smoking, alcohol abuse, and long-term work pressure, which have been widely associated with an elevated risk of TB according to other studies [22–25]. After the onset of TB, inadequate and prolonged treatment combined with drug misuse can result in the development of drug resistance within the body, further exacerbating the challenges associated with treatment. The proportion of ASIR, ASPR, ASR of DALYs, and ASDR increased with age, reflecting the higher burden of TB and MDR-TB in older individuals. Research conducted by Zhang Ting and colleagues [26] has indicated that the global burden of TB primarily affects the middle-aged and elderly population (aged 40–60). The prolonged use of anti-TB drugs, coupled with a natural decline in immune function as individuals age [27], often leads to a higher susceptibility to MDR-TB among the elderly. Therefore, in the prevention and control of MDR-TB, this study recommended intensifying screening efforts among TB patients to ensure early detection, timely diagnosis, and prompt treatment. TB patients should adhere to the principles of early, appropriate, combination, regular, and comprehensive medication, in order to prevent the progression to drug resistance. It is worth noting that our study provided a more in-depth analysis of population information compared to other studies [28, 29], specifically, we have compared the burden of MDR-TB in different age groups, taking into account the variations between sexes.

Regarding the region distribution of MDR-TB, our findings showed the top three cases were in India, China and Pakistan which was consistent with the WHO reports [4, 5, 30]. These regions have reported the highest incidence rates of TB, which can be attributed to the ecnomics and undernutrition [1, 4, 5], certainly, other social determinants should be taken into consideration as well, such as education, medical techniques, occupation, and social class [31, 32]. However, when we included the total population of the country for burden estimation, we discovered that the Sub-Saharan Africa, particularly in Somalia, had a significantly larger rate compared to other regions. Although the number of cases in this region was not obvious, the proportion of patients in relation to the total population of their country was significant, which would impose a serious burden on their country. Other related study [26] have also indicated that, there were higher mortality rates due to TB in the eastern and western regions of Sub-Saharan Africa in 2019. This finding highlights the urgent need to pay attention to the issue of MDR-TB in the African region as well. A recent study has demonstrated that the implementation of effective social protection and poverty eradication programs could potentially lead to a substantial reduction in the incidence and mortality rates of TB [33]. Hence, it is imperative for these countries to prioritize the allocation of healthcare resources, particularly focusing on impoverished regions, reducing hunger, and developing social protection programs specifically targeting underprivileged households with TB patients.

In addition, the Joinpoint regression analysis provides temporal trends rather than the overall percentage change trends from 1990 to 2019, which offers more accurate and detailed information than others [34, 35]. All four indicators exhibited an overall ascending tendency from 1990 to 2019, with a subsequent decline from 2005 to 2019. This trend was consistent with the global tuberculosis report [5], which indicated a decline in the number of deaths and incidence rate of TB but not at a rate sufficient to achieve the 2020 milestone of a 20% reduction. Meanwhile, Lange emphasized that despite the global decrease in the burden of TB and improvements in treatment rates, MDR-TB remains a substantial global health threat, characterized by high mortality rates [36]. Therefore, within the field of TB prevention and control, it is essential to recognize MDR-TB as a critical component. The focus should not only be on reducing the incidence of TB but also on efforts to decrease the number of drug-susceptible TB cases transitioning to drug-resistant forms.

Some limitations should be acknowledged. The data used in this study, derived from GBD 2019, provides estimated values that depend on the quality of the underlying data, which may vary across regions, leading to potential underestimations of the burden of MDR-TB in certain areas. Currently, the GBD dataset is only updated until the year 2019, and the findings of this study exclusively examine the prevalence of MDR-TB infections up to and including 2019. Further research is warranted to investigate the post-2019 trends in MDR-TB prevalence and the impact of the coronavirus disease 2019 pandemic on MDR-TB dynamics. Furthermore, this study did not conduct specific analyses of countries with high MDR-TB burdens, which would require further investigation.

## Conclusion

In conclusion, our analysis of GBD 2019 data has revealed significant disparities in the global distribution of MDR-TB, encompassing variations in population, regions, and temporal. It is evident that the MDR-TB stayed on a persistent threat to public health, and concerted global action is necessary to address this challenge. Countries and health organizations should adopt a multi-faceted approach tailored to their specific circumstances, aiming to identify suitable policies and strategies to collectively work towards the goal of “Ending TB”.

### Electronic supplementary material

Below is the link to the electronic supplementary material.

Supplementary Material 1

Supplementary Material 2

## Data Availability

The datas used were publicly for this study. The website of the data is: https://vizhub.healthdata.org/gbd-results/.

## Abbreviations

MDR-TB: multidrug-resistant tuberculosis
Mtb: Mycobacterium tuberculosis
HIV: human immunodeficiency virus
AIDS: acquired immune deficiency syndrome
UIs: uncertainty intervals
ASIR: age-standardized incidence rate
ASPR: age-standardized prevalence rate
ASR of DALYs: age-standardized disability-adjusted life years rate
ASDR: age-standardized death rate
APC: annual percentage change
AAPC: average annual percentage change
CI: confidence interval
