# Supplementary material for: Global prevalence and burden of multidrug-resistant tuberculosis from 1990 to 2019
Source: BMC Infect Dis. 2024 Feb 22;24:243. doi: 10.1186/s12879-024-09079-5 (PMC10885623; doi:10.1186/s12879-024-09079-5)
Supplement: Supplementary file 1 — Supplementary Material 1 [file 12879_2024_9079_MOESM1_ESM.docx]

Multidrug-resistant tuberculosis, extensively drug-resistant tuberculosis, and drug- susceptible tuberculosis


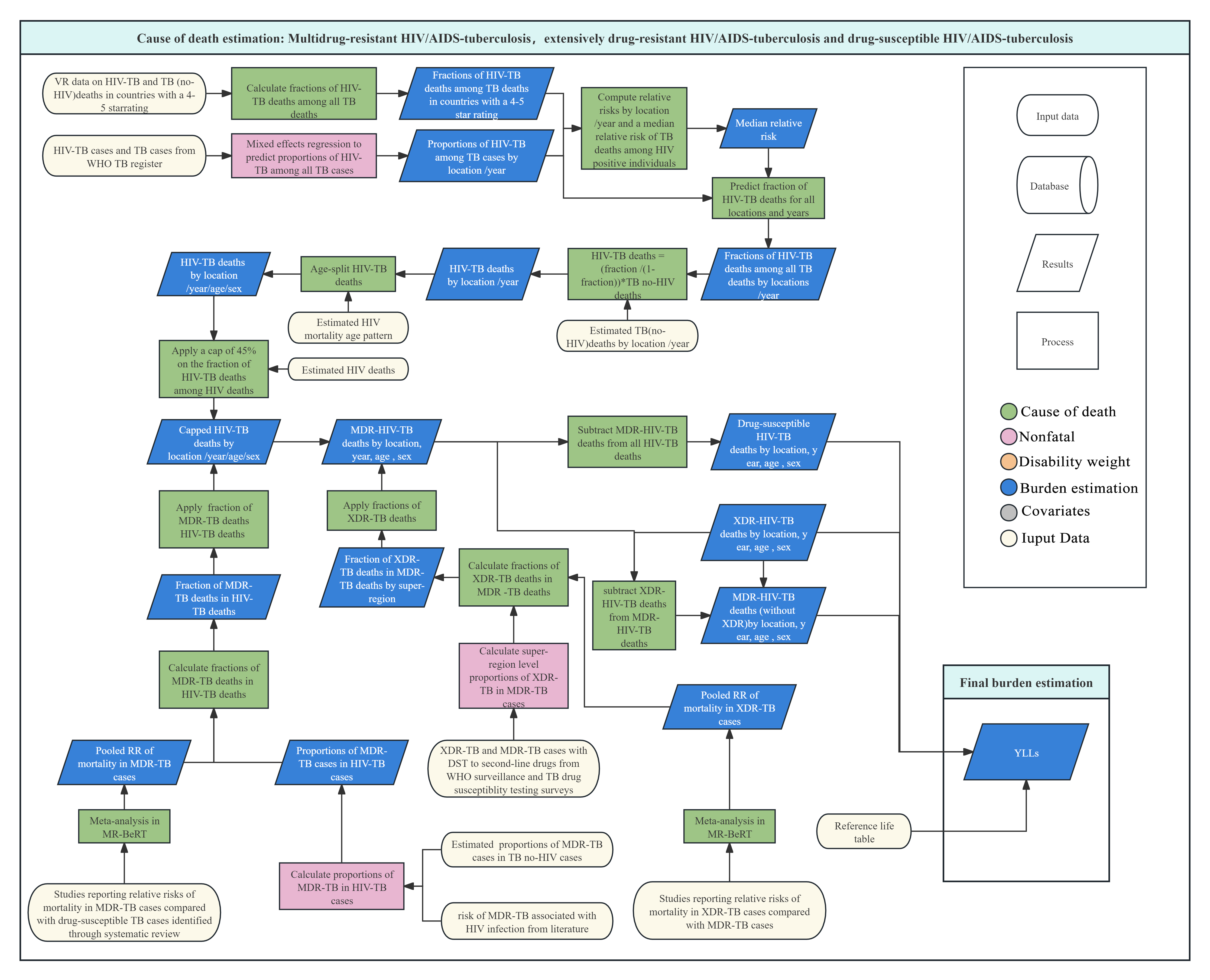


Input data

Input data include: (i) the number of drug-resistant cases by type (multidrug-resistant tuberculosis [MDR-TB], extensively drug-resistant tuberculosis [XDR-TB], all TB cases with a drug-susceptible testing [DST] result for isoniazid and rifampicin, and MDR-TB cases with DST for second-line drugs) from routine surveillance and surveys reported to the World Health Organization, (ii) data from studies (identified through our systematic review) reporting on the relative risk of death in MDR-TB cases compared with non-MDR TB (drug-susceptible TB) cases, and the relative risk of death in XDR-TB cases compared with MDR-TB cases, and (iii) the risk of MDR-TB associated with HIV infection from the literature.^1^

PRISMA diagram of MDR-TB mortality relative risk in GBD 2019

Records identified through

database searching

(n=97)

Additional records identified

through other sources

(n=0)

Records screened

(n=97)

Records excluded

(n=60)

Full-text articles excluded (n=27)

Foreign language articles not assessed

(n=0)

Full-text articles assessed

for eligibility

(n=37)

Studies included in meta-

analysis

(n=10)

Modelling strategy

We conducted a systematic review and meta-analysis of studies reporting the relative risk of death in MDR-TB cases compared with drug-susceptible TB cases. We ran spatiotemporal Gaussian process regressions to predict the proportions of new TB cases with MDR-TB, proportions of retreated TB cases with MDR-TB, and proportions of retreated cases among all TB cases for all locations and years. We also calculated the proportions of new TB cases among all TB cases. We then computed the weighted average of the proportions of new and retreated cases with MDR-TB at the 1000-draw level. We then used the weighted average proportions of MDR-TB, along with the HIV-TB and TB no-HIV incidence estimates (from our modelling of non-fatal TB), and the relative risk of MDR-TB associated with HIV infection from the literature[1] to compute the proportions of MDR-TB cases among HIV-negative TB cases (P_MDRnoHIVc,y,a,s_) by location, year, age, and sex using the following formula:

$$P_{MDRnoHIVc,y,a,s}=\frac{MDR_{c,y}}{\left( 1+\left( RR_{HIV}\frac{HIVTB_{c,y,a,s}}{TBnoHIV_{c,y,a,s}} \right) \right)TBnoHIV_{c,y,a,s}}$$

where MDR*_c,y_* is the number of all MDR-TB cases among HIV-positive and HIV-negative individuals by location and year, RR*_HIV_* is the relative risk of MDR-TB associated with HIV infection, *HIVTB_c,y,a,s_* is the number of HIV-TB incident cases by location, year, age, and sex, and *TBnoHIV_c,y,a,s_* is the number of TB no-HIV incident cases by location, year, age, and sex. We then computed the fraction of MDR-TB deaths among all HIV-negative TB deaths (*𝐷_MD RnoHIV c,y,a,s_*) using the following formula:

$$D_{MDRnoHIVc,y,a,s}=\frac{P_{MDRnoHIVc,y,a,s}RR_{MDR}}{P_{MDRnoHIVc,y,a,s}RR_{MDR}+1-P_{MDRnoHIVc,y,a,s}}$$

where 𝑅𝑅*_MDR_* is the relative risk of death in MDR-TB cases compared with drug-susceptible TB cases. In GBD 2019, the pooled relative risk was derived from a meta-analysis in the meta-regression with Bayesian priors, regularization, and trimming (MR-BRT) model. After derivation of the pooled relative risk, we then applied the predicted HIV-MDR-TB death fractions to all HIV-TB death estimates to generate HIV-MDR-TB deaths by location, year, age, and sex. Next, we subtracted MDR-TB deaths from all TB deaths to generate drug-susceptible TB deaths by location, year, age, and sex.

To separate out XDR-TB from MDR-TB, we aggregated the XDR-TB cases and MDR-TB cases (with DST for second-line drugs) up to the super-region level and calculated the super-region-level proportions of XDR-TB among MDR-TB cases. Next, we computed the super-region-specific fractions of XDR-TB deaths among all MDR-TB deaths (𝐷*_XDRsr_*) using the following formula:

$$D_{XDRsr}=\frac{P_{XDRsr}RR_{XDR}}{P_{XDRsr}RR_{XDR}+1-P_{XDRsr}}$$

where P*_XDRsr_* is the proportion of XDR-TB among MDR-TB cases for each super-region, and 𝑅𝑅_XDR_ is the pooled relative risk of mortality in XDR-TB cases compared with MDR-TB cases. Similar to the pooled relative risk for MDR-TB, the derivation of the pooled relative risk of mortality in XDR-TB was computed

with a meta-analysis in the MR-BRT model for GBD 2019. These fractions were then applied to MDR-TB deaths in corresponding countries within the super-regions to produce XDR-TB deaths by location, age, and sex for the most recent year of estimation. We linearly extrapolated XDR-TB mortality rates back, assuming the mortality rates were zero in 1992, one year before 1993 when XDR-TB was first recorded in USA surveillance data.[2] Finally, we subtracted XDR-TB deaths from MDR-TB deaths to generate MDR-TB (without extensive drug resistance) deaths by location, year, age, and sex.

**References:**

1. Mesfin YM, Hailemariam D, Biadglign S, Kibret KT. Association between HIV/AIDS and multi-drug resistance tuberculosis: a systematic review and meta-analysis. PLoS One. 2014;9(1):e82235.

2. Centers for Disease Control and Prevention (CDC). Extensively Drug-Resistant Tuberculosis --- United States, 1993—2006. MMWR. 2007; 56(11);250-253
