# Supplementary figures and images for: Global prevalence and burden of multidrug-resistant tuberculosis from 1990 to 2019
Source: BMC Infect Dis. 2024 Feb 22;24:243. doi: 10.1186/s12879-024-09079-5 (PMC10885623; doi:10.1186/s12879-024-09079-5)

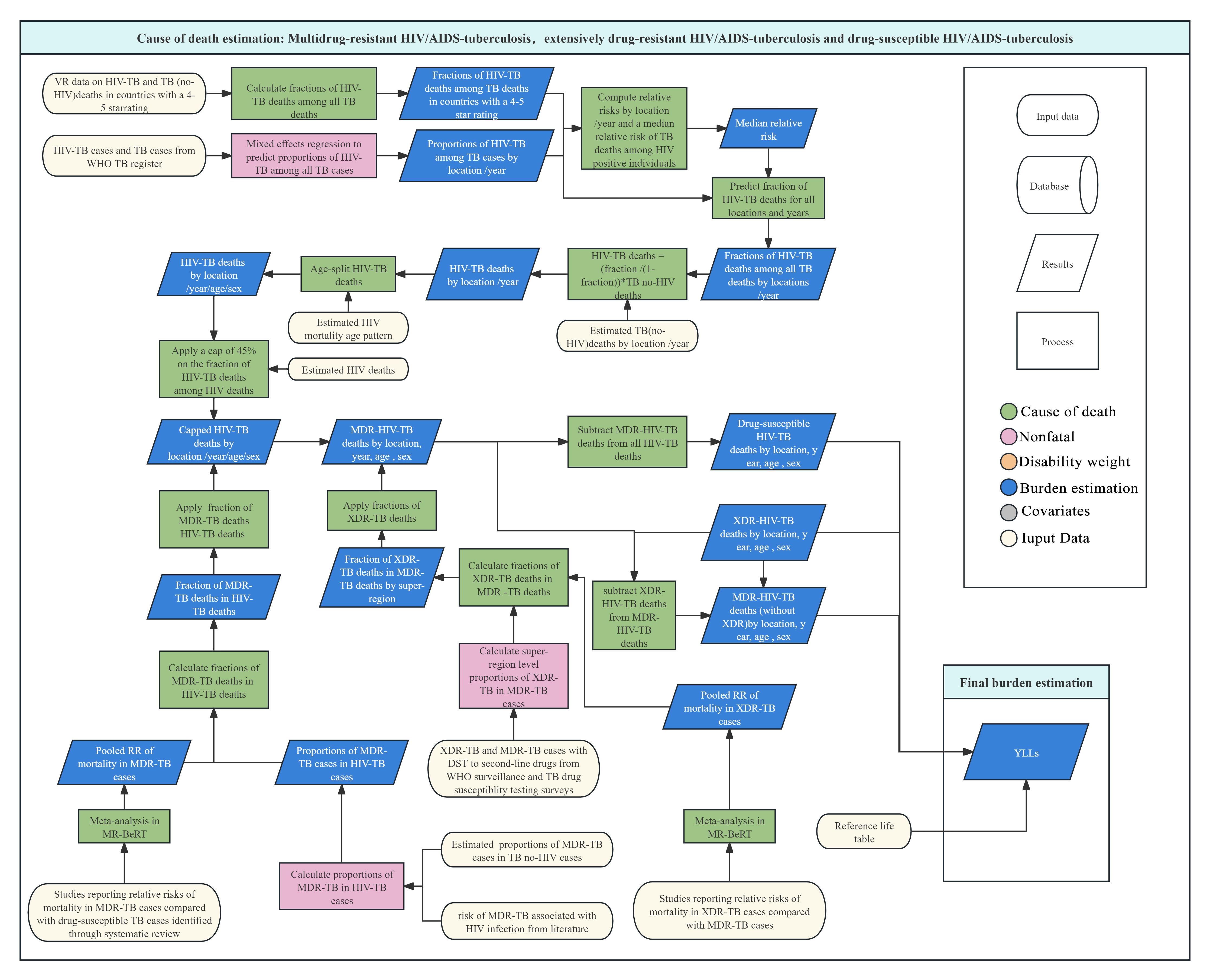

Supplement: Supplementary file 2 — Supplementary Material 2 [file 12879_2024_9079_MOESM2_ESM.jpg]
